# Comparing determinants of alien bird impacts across two continents: implications for risk assessment and management

**DOI:** 10.1002/ece3.1144

**Published:** 2014-06-23

**Authors:** Thomas Evans, Sabrina Kumschick, Ellie Dyer, Tim Blackburn

**Affiliations:** 1 Imperial College London, Silwood Park CampusBuckhurst Road, Ascot, Berkshire, SL5 7PY, U.K; 2 Centre for Invasion Biology, Department of Botany and Zoology, Stellenbosch University, Private Bag X1Matieland, 7602, South Africa; 3 Institute of Zoology, Zoological Society of London, Regent's ParkLondon, NW1 4RY, U.K; 4 Centre for Biodiversity and Environment Research, Department of Genetics, Evolution and Environment, University College LondonLondon, WC1E 6BT, U.K; 5 Distinguished Scientist Fellowship Program, King Saud UniversityRiyadh, 1145, Saudi Arabia; 6 Environment Institute, School of Earth & Environmental Sciences, University of AdelaideAdelaide, South Australia, 5005, Australia

**Keywords:** Alien birds, biological invasion, habitat generalism, impact prediction, life history traits, risk assessment

## Abstract

Invasive alien species can have serious adverse impacts on both the environment and the economy. Being able to predict the impacts of an alien species could assist in preventing or reducing these impacts. This study aimed to establish whether there are any life history traits consistently correlated with the impacts of alien birds across two continents, Europe and Australia, as a first step toward identifying life history traits that may have the potential to be adopted as predictors of alien bird impacts. A recently established impact scoring system was used in combination with a literature review to allocate impact scores to alien bird species with self-sustaining populations in Australia. These scores were then tested for correlation with a series of life history traits. The results were compared to data from a previous study in Europe, undertaken using the same methodology, in order to establish whether there are any life history traits consistently correlated with impact across both continents. Habitat generalism was the only life history trait found to be consistently correlated with impact in both Europe and Australia. This trait shows promise as a potential predictor of alien bird impacts. The results support the findings of previous studies in this field, and could be used to inform decisions regarding the prevention and management of future invasions.

## Introduction

Alien bird species can cause significant and wide-ranging environmental and economic damage, including competition with native species for habitat and food, damage to agricultural crops and infrastructure, and impacts to human health and welfare through the spread of disease, fouling of buildings and noise disturbance (Long 1981; Brochier et al. 2010). The total economic loss resulting from the impacts of alien birds in just six countries (UK, USA, Australia, South Africa, India and Brazil) has been estimated at US$ 2.4 billion per year (Pimentel 2002), while three bird species (European starling *Sturnus vulgaris*, common myna *Acridotheres tristis* and red-vented bulbul *Pycnonotus cafer*) are included on the IUCN list of 100 of the world's worst invaders (Lowe et al. 2004). In Australia, the common myna and European starling compete with native species for nest holes, potentially affecting the breeding success of the red-rumped parrot (*Psephotus haematonotus*), crimson rosella (*Platycercus elegans*) and eastern rosella (*Platycercus eximius*) (Pell and Tidemann 1997), while the mallard (*Anas platyrhynchos*) threatens the native Pacific black duck (*Anas superciliosa*) through hybridization (Tracey et al. 2008). The common blackbird (*Turdus merula*) and European starling are significant pests of Australian agriculture, causing damage to vineyards and orchards (Tracey and Saunders 2003).

Given the enormous environmental and economic costs of alien species, and the fact that early interventions are often more cost-effective in their successful control (Pluess et al. 2012), it is clearly desirable to be able to predict the impacts of alien species prior to their establishment and invasion (Kumschick and Richardson 2013). One step toward prediction would be to identify potential determinants of impact in the form of the life history traits associated with damaging species, which can lead to a better understanding of the mechanisms behind impact.

Characteristics relating to the breadth and quantity of resources used by species have previously been identified as potential indicators of the magnitude of alien bird impacts. Habitat generalism (the number of broad habitats types that a species may occupy) is positively correlated with impact for alien bird species in Europe (Shirley and Kark 2009; Kumschick et al. 2013). Furthermore, bird species with large native breeding ranges tend to have higher impacts (Kumschick et al. 2013). This suggests that species with broad environmental tolerances have a greater opportunity to invade and colonize new regions than species with narrow habitat tolerances, and hence have greater opportunity to cause adverse impacts. Body mass has also been found to be positively correlated with impact (Kumschick et al. 2013), consistent with the proposition that species may have more severe impacts on the environment and economy if they have higher resource requirements. It has also been suggested that species producing multiple broods per year, and therefore having high population growth potential, have more significant impacts than species producing comparatively fewer broods (Shirley and Kark 2009).

However, these previous studies were restricted to the impacts of alien birds in Europe, and it is therefore not known if their results are consequences of the species introduced, or of peculiarities of the European environment. For example, the highly seasonal climate that prevails over much of Europe may make different demands on the tolerances of introduced species, if they are to be successful, than climates with less dramatic winter temperature lows. Conversely, broad environmental tolerances may promote success in all alien environments (cf Cassey et al. 2004; Sol et al. 2005; Blackburn et al. 2009). From a risk assessment and management perspective, an understanding of the generality of impact correlates is required to determine which life history traits have the potential to be reliable impact predictors.

Here, we use a recently established impact scoring system (Kumschick and Nentwig 2010; Nentwig et al. 2010) to explore correlates of impact in alien bird species in Australia. We used data from a previous study carried out in Europe (Kumschick et al. 2013) to compare relationships for Europe and Australia, as a first step in determining whether there are common correlates of impact associated with alien bird species on both continents. Given the findings of previous studies (Shirley and Kark 2009; Kumschick and Nentwig 2010; Kumschick et al. 2013), we predicted that large-bodied, habitat generalist alien bird species that are widespread in their native ranges should have the highest impacts when introduced in both Europe and Australia.

## Methods

### Quantifying impact

A review of published literature was undertaken in order to compile a list of alien bird species with self-sustaining populations in Australia. Key sources of information included the Global Invasive Species Database (GISD; http://www.issg.org/database/welcome/), Global Avian Invasions Atlas (GAVIA unpubl.), BirdLife Australia (http://birdlife.org.au), the Invasive Species Council of Australia (http://invasives.org.au) and the Invasive Species Cooperative Research Centre of Australia (http://www.invasiveanimals.com). For the purpose of the assessment, Australia was defined as the continent of Australia and the island state of Tasmania. Only species whose native distribution lies entirely outside of Australia were considered, in order to ensure that only true alien populations were included within the study. Alien bird species present in Australia, but without self-sustaining populations, were excluded from the assessment: a species was considered to have a self-sustaining population if records indicated its presence in Australia for over 25 years (following Gebhardt et al. 1996). Species that have naturally colonized Australia were also excluded from the assessment. We identified 27 species as fitting the above mentioned criteria (Data S1).

We used Web of Science to collate information on the reported impacts of the alien bird species selected. Searches were undertaken for each species using both the common and scientific species name. Targeted consultation was also undertaken with key organizations in order to gather grey literature. Key sources of information on impacts included Long (1981), and the websites of the Invasive Species Council of Australia (http://invasives.org.au), the Invasive Species Cooperative Research Centre of Australia (http://www.invasiveanimals.com), the Department of Agriculture, Fisheries and Forestry (http://www.daff.gov.au), the Office of Environment and Heritage (NSW) (http://www.environment.nsw.gov.au), the Department of Agriculture, Fisheries and Forestry (Queensland) (http://www.daff.qld.gov.au), the Department of Primary Industry and Regions South Australia (http://www.pir.sa.gov.au) and the Department of Agriculture and Food (Western Australia) (http://www.agric.wa.gov.au).

Following Kumschick and Nentwig (2010), impacts were categorized as either environmental or economic, and within these two categories were allocated to one of six subcategories. For environmental impacts the subcategories were herbivory, competition, predation, transmission of disease to wildlife, hybridization and ecosystem. For economic impacts the subcategories were agriculture, animal production, forestry, infrastructure, human health and human social life. Impact scores were then assigned to each species to quantify the magnitude of its impacts in each subcategory. Following a review of each publication containing information on the impacts of a species, a score between 0 and 5 was allocated for each subcategory (where 0 equates to no impact known or detectable, and 5 equates to the most severe impact possible in the respective category). Impact scores were therefore ordinal, with higher scores always equating to a higher impact. In order to minimize subjectivity, a series of written descriptions relating to each impact level (0–5) were used to guide the scoring process (Kumschick and Nentwig 2010; Data S2). To ensure objectivity, scoring was undertaken independently by two people, T.E. and S.K. Furthermore, previous studies have shown and discussed the robustness of the scoring approach (Nentwig et al. 2010; Kumschick and Nentwig 2011; Kumschick et al. 2013). The highest score allocated was taken as the impact score for each subcategory. This allowed for the potential worst-case scenario to be established in terms of potential impacts.

The overall environmental or economic impact of a species was established by summing the scores of all subcategories, and therefore took a value of between 0 and 30 for each of the environmental and economic subcategories. The total impact score per species was calculated by summing the environmental and economic impact scores, and therefore took a value of between 0 and 60. Total impact scores for each species are provided in Data S1. Impact measured in this way integrates two of the components (Abundance and per capita Effect) given in the classic Parker et al. (1999) equation for overall impact (Impact = Range × Abundance × per capita Effect; see also Kumschick et al. 2013; cf. Jeschke et al. 2014).

Impact scores for 26 alien bird species in Europe, calculated using the same methodology, were taken from Kumschick and Nentwig (2010). Three species are present on both continents: the California quail (*Callipepla californica*), wild turkey (*Meleagris gallopavo*) and common myna. However, since only impact records for the respective continent were taken into account in both data sets, the sources of data for the impact scores do not overlap.

### Life history traits

The magnitude of documented economic and environmental impacts, as well as their combination (hereafter called total impact) were compared to a set of species traits. As in Kumschick et al. (2013), species traits were selected to provide an indication of the breadth of resources that a species can use (diet breadth, habitat breadth and native breeding range size – the latter being an indicator of the range of habitats or environments a species may be able to occupy) and the amount of resources that a species is likely to use (body mass and clutch size).

For alien bird species in Australia, data on body mass (g) and native breeding range size (km^2^) were taken from Orme et al. (2005) and Olson et al. (2009), kindly supplied by those authors. Information on clutch size (number of eggs per brood) was taken from BirdLife International (http://www.birdlife.org) and the British Trust for Ornithology (BTO) (http://www.bto.org). Following Kumschick et al. (2013), diet breadth was estimated as the number of the following eight major food types consumed by a species: grasses/forbs; seeds/grains; fruits/berries; pollen/nectar/flowers; tree leaves/branches/bark; roots/tubers; invertebrate prey; vertebrate prey/carrion. Habitat breadth was estimated as being the number of the following ten habitat categories included in a species native range: marine habitats, including littoral rock and sediment; coastal habitats; inland surface waters; mires, bogs, and fens; grasslands and lands dominated by forbs, mosses or lichens; heathland, scrub, and tundra; woodland, forest, and other wooded land; inland unvegetated or sparsely vegetated habitats; regularly or recently cultivated agricultural, horticultural, and domestic habitats; constructed, industrial, and other artificial habitats. Information on diet and habitat breadth was taken from BirdLife International and the BTO. Data on the same life history traits for alien birds in Europe were taken from Kumschick et al. (2013), and mainly derived from the same sources as for the species in Australia.

### Analysis

All analyses were carried out using R (version 3.0.2) (R Development Core Team 2008). The impact scores allocated by each assessor (T.E. and S.K.) were compared using Pearson's product-moment correlation. Comparisons of the three impact measures (environmental, economic and total) between the two continents were undertaken separately using Wilcoxon rank sum tests. The relationship between each of the life history traits and the environmental, economic and total impacts of alien birds was assessed using generalized linear mixed effects models with Poisson errors using the lme4 package (Bates et al. 2012). A random effect for family was included to account for potential autocorrelation among species due to their phylogenetic relatedness. Models included a fixed effect of region (Australia or Europe) to test for different relationships between alien bird impacts and traits in different regions, together with an interaction term between region and the trait concerned. If the interaction term was significant, separate analyses of the European and Australian data sets were undertaken to explore the relationships further. We analyzed each life history trait separately to avoid issues of over-fitting given our relatively small sample sizes. We did not employ formal methods to correct for multiple tests but were careful not to over-interpret relationships with *P* values > 0.01. Data for two variables (body mass and native breeding range size) were log transformed for analysis. Impact scores from Kumschick et al. (2013) were analyzed using the same methodology, and hence we recovered the same results for European bird impacts as in that paper.

## Results

Total impact scores allocated by each reviewer were 611 (T.E.) and 595 (S.K.). The percentage of the possible maximum impact score allocated was 14.14% (T.E.) and 13.77% (S.K.). There was an extremely high correlation between the impact scores allocated by the two reviewers (*r* = 0.99, df = 186, *P* < 0.001).

More than half of the alien bird species found in Australia (14 out of 27) were passeriformes, with the remainder consisting of galliformes (6), columbiformes (4), anseriformes (2) and struthioniformes (1). The majority of the alien bird species found in Europe were either anseriformes (9 of 26) or galliformes (9), with the remainder consisting of passeriformes (4), psittaciformes (2), phoenicopteriformes (1) and ciconiiformes (1). More than half of the species found in Australia (14 of 27) are native to Europe. Conversely, only one alien species found in Europe, the black swan (*Cygnus atratus*), is native to Australia. Of the three alien species present in both Europe and Australia, the California quail and wild turkey had no recorded impacts on either continent, while the common myna had greater environmental and economic impacts in Australia (total impact score = 27) than in Europe (total impact score = 3).

There was no detectable difference in total impact scores between the two continents (Wilcoxon rank sum test, *W* = 344, *P* = 0.91). The average total impact score for Australia was 6.70 (of a maximum score of 60), with the highest total impact score being 27 for the common myna. The average total impact score for Europe was 5.31, with the highest total impact score being 36 for the Canada goose (*Branta canadensis*) (Kumschick and Nentwig 2010). Economic impacts were higher in Australia (Wilcoxon rank sum test, *W* = 253, *P* = 0.05), but we found no difference between environmental impact scores on the two continents (*W* = 435, *P* = 0.12).

In Europe, environmental impacts were found to be greater than economic impacts (Wilcoxon rank sum test, *W* = 478, *P* = 0.006), while in Australia, no difference was found between environmental and economic impacts (Wilcoxon rank sum test, *W* = 320.5, *P* = 0.42). We found no difference when comparing combined environmental impacts for Australia and Europe with combined economic impacts for the two continents (Wilcoxon rank sum test, *W* = 1603.5, *P* = 0.18).

**Figure 1:**
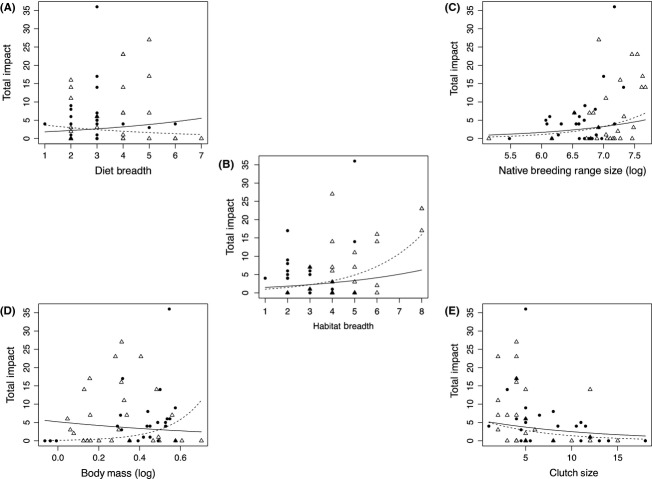
The relationship between total impact and each predictor variable for alien bird species in Europe (filled circles and dashed line) and Australia (unfilled triangles and solid line). Maximum possible impact score per species = 60. The fitted curves are calculated from the parameters of the mixed models given in Table 1. (A) = Diet breadth; (B) = Habitat breadth; (C) = Native breeding range size (log); (D) = Body mass (log); (E) = Clutch size.

The three variables that relate to breadth of resource use (diet breadth, habitat breadth and native breeding range size) were all correlated with total impact (Table 1). However, different relationships between diet breadth and impact were identified for the two continents, as indicated by the interaction term in the relevant model (Table 1). Further analysis for the two continents separately showed that there was no significant relationship between diet breadth and total impact in Europe, but that total impact was positively related to diet breadth in Australia (Table 2; Fig. 1). No relationships were identified between total impact and the two variables relating to the potential magnitude of resource use (body mass and clutch size). However, a significant interaction term was noted for body mass (Table 1): a positive correlation between total impact and body mass was found in Europe, but no relationship was found in Australia (Table 2; Fig. 1).

**Table 1:** The relationships between environmental, economic and total impact metrics and the predictor variables (listed in the first column). All the parameters in this table derive from generalized linear mixed effects models with Poisson errors using the lme4 package (Bates et al. 2012). Models included a main effect of the predictor variable, a fixed effect of region (Australia or Europe), and a term for the interaction between region and the trait concerned. Total sample size = 53 (26 species alien to Europe, 27 species alien to Australia)

	Environmental impact			Economic impact			Total impact		
			
	Est	Std Error	*P*	Est	Std Error	*P*	Est	Std Error	*P*
Diet breadth									
Main effect	0.04	0.14		0.21	0.10	*	0.18	0.09	*
Fixed effect	0.80	0.71		0.56	0.74		1.08	0.50	*
Interaction	−0.17	0.17		−0.45	0.20	*	−0.39	0.12	**
Habitat breadth									
Main effect	0.24	0.08	**	0.18	0.05	***	0.20	0.05	***
Fixed effect	1.13	0.60	.	−4.14	0.84	***	−0.58	0.41	
Interaction	−0.14	0.14		0.94	0.19	***	0.19	0.10	.
Native breeding range size (log)									
Main effect	0.61	0.42		0.70	0.35	*	0.66	0.27	*
Fixed effect	0.35	3.64		−21.97	5.93	***	−3.09	2.79	
Interaction	−0.13	0.52		3.08	0.84	***	0.44	0.40	
Body mass (log)									
Main effect	−2.12	1.42		2.45	2.22		−1.08	1.42	
Fixed effect	−2.14	0.78	**	−8.79	2.31	***	−3.64	0.80	***
Interaction	5.73	1.76	**	15.80	4.34	***	7.35	1.67	***
Clutch size									
Main effect	−0.08	0.06		−0.10	0.06	.	−0.08	0.04	.
Fixed effect	0.52	0.48		0.15	0.66		0.03	0.38	
Interaction	−0.04	0.07		−0.22	0.11	*	−0.06	0.06	

Est = Estimated Coefficient; Std Error = Standard Error; ***P < 0.001; **P < 0.01; *P < 0.05; *P < 0.1.

**Table 2:** The relationship between environmental, economic, and total impact metrics and the predictor variables (listed in the first column) for the European and Australian data sets separately, in those cases where the interaction term for the combined data set (Table 1) indicated a significant difference in the slope for the two data sets. All the parameters in this table derive from generalized linear mixed effects models with Poisson errors using the lme4 package (Bates et al. 2012)

	Environmental impact			Economic impact			Total impact		
			
	Est	Std Error	*P*	Est	Std Error	*P*	Est	Std Error	*P*
Diet breadth									
Europe				−0.04	0.16		−0.06	0.09	
Australia				0.17	0.10		0.18	0.09	*
Habitat breadth									
Europe				1.08	0.16	***			
Australia				0.17	0.05	**			
Native breeding range size (log)									
Europe				3.36	0.71	***			
Australia				0.88	0.37	*			
Body mass (log)									
Europe	4.03	1.44	**	19.98	6.02	***	5.22	1.68	**
Australia	−1.93	1.70		1.49	2.07		0.13	1.71	
Clutch size									
Europe				−0.36	0.10	***			
Australia				−0.01	0.07				

Sample size = 26 (Europe) or 27 (Australia). Est = Estimated Coefficient; Std Error = Standard Error; ***P < 0.001; **P < 0.01; *P < 0.05; *P < 0.1.

**Figure 2:**
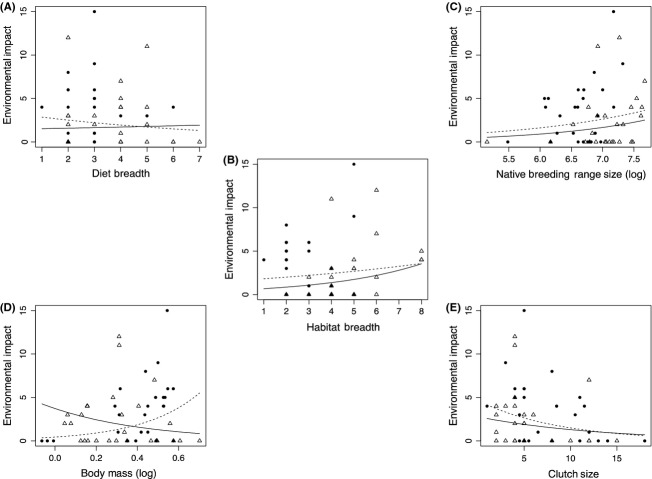
The relationship between environmental impact and each predictor variable for alien bird species in Europe (filled circles and dashed line) and Australia (unfilled triangles and solid line). Maximum possible impact score per species = 30. The fitted curves are calculated from the parameters of the mixed models given in Table 1. (A) = Diet breadth; (B) = Habitat breadth; (C) = Native breeding range size (log); (D) = Body mass (log); (E) = Clutch size.

The only variable correlated with environmental impact was habitat breadth (Table 1). Although no significant correlations were identified for the other predictor variables, a significant interaction term was noted for body mass (Table 1). Further analysis for the two continents separately identified a positive correlation between environmental impact and body mass in Europe but not Australia (Table 2; Fig. 2).

**Figure 3:**
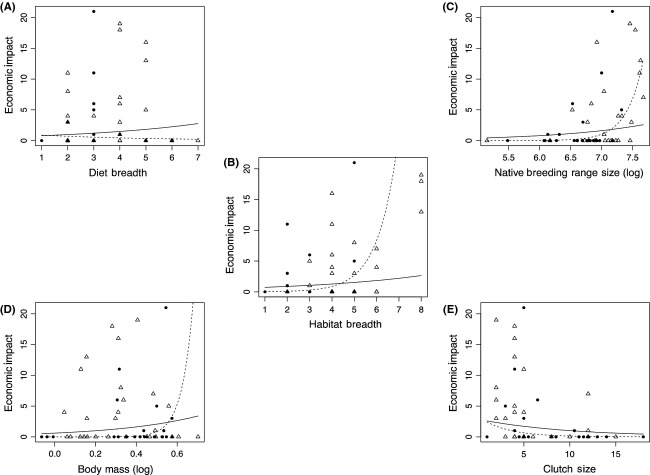
The relationship between economic impact and each predictor variable for alien bird species in Europe (filled circles and dashed line) and Australia (unfilled triangles and solid line). Maximum possible impact score per species = 30. The fitted curves are calculated from the parameters of the mixed models given in Table 1. (A) = Diet breadth; (B) = Habitat breadth; (C) = Native breeding range size (log); (D) = Body mass (log); (E) = Clutch size.

The same variables found to be significantly related to total impact were also correlated with economic impact (diet breadth, habitat breadth and native breeding range size) (Table 1). However, significant interaction terms were identified for all life history traits tested, indicating that the relationship with impact varied between continents for all variables. The relationships for habitat breadth and native breeding range size were significantly positive on both continents, but differed significantly in slope. Both variables are more steeply correlated with economic impact in Europe than in Australia (Table 2; Fig. 3).

## Discussion

As noted by Kumschick et al. (2013), the usefulness and predictive power of scoring systems of the type used here to quantify alien bird impacts has been demonstrated in numerous studies (e.g., Pheloung et al. 1999; Purvis et al. 2000; Fritz et al. 2009), and such approaches have been used to inform risk assessments (Leung et al. 2012). The scoring process used here is semi-quantitative and so is inevitably open to a certain degree of subjectivity. However, the use of the written impact descriptions to guide the allocation of impact scores, plus the relatively clear separation between the levels of impact represented by different levels within each scenario, helps to maintain an objective scoring process (see Data S2). Moreover, the high correlation between the impact scores allocated by T.E. and S.K. (*r* = 0.99) suggests that the scores allocated by the two reviewers were similar, supporting the assertion that use of the written descriptions maintained an objective scoring process.

To date, many risk assessments for alien species do not consider impact as part of the assessment process (e.g. border control risk assessment for vertebrates in Australia, Bomford 2008), and the majority of schemes do not include impact magnitude (Leung et al. 2012; but see Blackburn et al. 2014). Those that do consider impact often rely solely on invasion history elsewhere to determine the risk of impact in a new range (Kumschick and Richardson 2013). With increasing international trade and, as a consequence, increasing incidence of new alien invasions (Hulme 2009), for which we often have no data on invasion history elsewhere, it is important that we develop a range of indicators and metrics to predict potential impacts that do not rely on invasion history data (Ricciardi 2003.). In this regard, it would be helpful to be able to identify characteristics of species that relate to their impacts. Such relationships would potentially allow the impacts of species without alien populations to be predicted on the basis of their possession (or otherwise) of the relevant characteristics.

Previous studies on the impacts of alien bird species in Europe have found that traits relating to habitat generalism are the most consistent correlates of their environmental and economic impacts (Shirley and Kark 2009; Kumschick et al. 2013). We find the same result for the impacts of alien bird species in Australia. On both continents, environmental, economic and total impacts were higher for species that can occupy a broad range of habitats. Indeed, habitat generalism was the only trait we tested that was significantly correlated to all three measures of alien bird impact in Australia. Combined with the results of studies in Europe, this suggests that habitat generalism shows promise as a general predictor of impact for bird species. Furthermore, habitat generalism has been found to be correlated with impact and establishment success of alien mammals in Europe (Sol et al. 2008; Nentwig et al. 2010), and is also consistently related to establishment success for alien birds (Cassey et al. 2004; Sol et al. 2005; Blackburn et al. 2009): habitat generalists are both more likely to colonize new environments, and also to have detrimental impacts once established. We also found significant positive effects on total and economic impacts of a species' diet breadth and native geographic range size, both of which further suggest that alien bird impacts in Australia are determined by the breadth of resources a species may use. Native range size, but not diet breadth, is also correlated with alien bird impacts in Europe (Kumschick et al. 2013).

In contrast, traits relating to the amount of resources a species may use (body mass and clutch size) do not relate to the potential impact of a species. However, these metrics of resource use by species are per capita measures, and different conclusions might have been drawn had we had data on alien species abundance to factor into the analysis. For example, it is typically the case that small-bodied species attain higher densities and population sizes (Gaston and Blackburn 2000), and so may have an impact through weight of numbers rather than the magnitude of individual consumption. As noted above, several of the species known to have large economic impacts are small-bodied passerine species that can attain high population densities (e.g. European starling, common myna, red-whiskered bulbul), although these species also tend to have broad habitat preferences and large native geographic range sizes.

The total impacts recorded for alien bird species did not differ significantly between Australia and Europe, but recorded economic impacts were higher in Australia. In particular, Australia has suffered severe impacts to its agricultural industry as a result of several pest bird invasions (Tracey et al. 2007). Scores in the economic impact sub-category for ‘agriculture’ were greater than 3 (impact description: ‘Damage through feeding on crops, occasional threat to stored food, damage exceeds impact of the native fauna’) for two species in the European data set, but for seven species within the Australian data set. Problem species for Australian agriculture include the common blackbird (Department of Employment, Economic Development and Innovation (Queensland) 2010a); house sparrow (*Passer domesticus*) (Department of Agriculture and Food (Western Australia) 2000), common myna (Department of Agriculture and Food (Western Australia) 2008a) and European starling (Department of Agriculture and Food (Western Australia) 2007). A recent risk assessment estimated that should the European starling become established in Western Australia, it could cause crop damage costing AU$ 80 million per annum (ACIL Tasman Pty Ltd 2006). These passerine species are noted consumers of fruit (Tracey et al. 2007), highly mobile, and several species tend to form large flocks, suggesting that agricultural impacts may be related to abundance.

Scores for the economic impacts of alien birds were found to be nearly twice as high as the environmental impact scores in Australia. This difference could be due to more frequent reporting of economic impacts when compared to environmental impacts. The financial implications associated with many economic impacts means that those affected by an alien bird species often have a vested interest in reporting and addressing these impacts (e.g. crop damage). Economic impacts are directly linked to human concerns (e.g. impacts on infrastructure, property and agriculture), and are therefore more likely to be noticed and reported (and possibly perceived as being more severe) than many environmental impacts such as competition and predation, which are often less easy to detect or prove, and less of an immediate concern to the general public. Of course, economic and environmental impact scores measure very different quantities, and may not be directly comparable. Conversely, economic impacts were lower than environmental impacts in Europe (and lower also than economic impacts in Australia). This is not because of a greater awareness of environmental impacts in Europe, but rather because there are very few records of alien bird species having severe economic impacts in Europe. Indeed, 21 of the 26 birds from the European data set had economic impact scores of only 0 or 1 out of 30, with only two species scoring over 10 (Canada goose and ring-necked parakeet *Psittacula krameri*). Why the relative magnitudes of environmental and economic impacts of alien birds differ between the continents in this way would benefit from further investigation.

Of the three species found on both continents, only the common myna was found to have recorded impacts, and these impacts were observed to be greater in Australia. This is most likely to due to the fact that the common myna prefers warmer climates, being most abundant in tropical, subtropical and warm temperate areas (Department of Employment, Economic Development and Innovation (Queensland) 2009a). As such, Australia provides a greater area and range of suitable habitats for this species than Europe. In Europe, the common myna is restricted to several islands in the Atlantic Ocean, including the Canary Islands and St Helena, and parts of northern France (ISSG 2013). While our measures of impact do not incorporate the range size element of Parker et al.'s (1999) classic impact equation, nevertheless, the opportunities for impact by the common myna may currently be considerably lower in Europe because of the unsuitability of the climate. This may of course change if, as expected, the European climate changes significantly in the coming decades.

There are a number of alien bird species that pose a threat of invasion in Australia which possess traits associated with habitat generalism. A prime example is the house crow *(Corvus splendens)*, which has been transported to Australia on ships from Asia on numerous occasions. It is a habitat generalist and has a large native breeding range. Given that this species has significant impacts on both the environment (competing with, and preying on, native species) and the economy (the house crow is a major pest of agriculture, and a nuisance pest to people through noise, fouling and damage to infrastructure), this is precisely the type of species that should be monitored carefully (Department of Agriculture and Food (Western Australia) 2008b; Department of Employment, Economic Development and Innovation (Queensland) 2010b). Another example is the red-billed quelea (*Quelea quelea*), which is kept as an exotic pet in Australia. This species also has a large native breeding range, and given that it can form nomadic super-colonies of up to 30 million individuals, and is a significant pest to agriculture, causing damage to grain crops in its alien range costing approximately US$ 70 million per annum, the red-billed quelea should also be monitored with care (Department of Employment, Economic Development and Innovation (Queensland) 2009b).

This study has identified life history traits that show potential to explain trends associated with alien bird impacts in two studies on two continents carried out using the same methodology. The results support the findings of previous studies, which also found a correlation between habitat generalism and impact (Shirley and Kark 2009; Kumschick et al. 2013). This suggests that habitat generalism shows promise as a general predictor of impact for alien bird species more widely than just in Europe and Australia. The traits identified in this study should now be further tested as actual predictors of impact by applying the methodology adopted in this study to other regions and taxa.

## Conclusions

As noted by Ricciardi et al. (2013), our ability to predict the impacts of alien species has been limited by the lack of a theoretical framework from which to test hypotheses on species impacts. They suggest that a consideration of life history traits could form one aspect of this framework. Encouragingly, this study provides evidence that the metric used, as developed by Nentwig et al. (2010) and further refined by Kumschick and Nentwig (2010), can be effectively applied in different countries and continents (Europe and Australia). It therefore provides an excellent platform from which to produce directly comparable studies with regards to the impacts of a wide range of alien species both regionally and globally. By identifying consistent impact correlates for several impact measures on two continents, we have come one step closer to predicting the impact of alien birds. However, additional work should be undertaken in other countries and regions, and with a broader range of life history traits, to test the predictive (as opposed to explanatory) power of the variables, and to explore whether there are regional and global patterns associated with the traits of alien birds and their environmental and economic impacts.

## Supporting Information

**Data S1.** Environmental and economic impact scores for 27 alien bird species with self-sustaining populations in Australia. **Data S2.** Written descriptions of impact levels for each environmental and economic sub-category, as published by Kumschick and Nentwig (2010).
