# Supplementary material for: Comparing determinants of alien bird impacts across two continents: implications for risk assessment and management
Source: Ecol Evol. 2014 Jun 23;4(14):2957–67. doi: 10.1002/ece3.1144 (PMC4130451; doi:10.1002/ece3.1144)
Supplement: Supplementary file 1 [file ece30004-2957-SD1.docx]

**Supplementary Dats S1:** Environmental and economic impact scores for 27 alien bird species with self-sustaining populations in Australia. Total impact scores for each species are the sum of the environmental and economic impact scores.

| Order | Family | Species (scientific name) | Species (common name) | Environmental impact score | Economic impact score |
| --- | --- | --- | --- | --- | --- |
| *Anseriformes* | *Anatidae* | *Anas platyrhynchos* | Mallard | 7 | 7 |
|  |  | *Cygnus olor* | Mute swan | 0 | 0 |
| *Columbiformes* | *Columbidae* | *Columba livia* | Rock dove | 4 | 19 |
|  |  | *Streptopelia chinensis* | Spotted turtle-dove | 3 | 8 |
|  |  | *Streptopelia risoria* | Barbary dove | 1 | 6 |
|  |  | *Streptopelia senegalensis* | Laughing turtle-dove | 0 | 3 |
| *Galliformes* | *Numididae* | *Numida meleagris* | Helmeted guineafowl | 0 | 0 |
|  | *Odontophoridae* | *Callipepla californica* | California quail | 0 | 0 |
|  | *Phasianidae* | *Gallus gallus* | Red junglefowl | 0 | 0 |
|  |  | *Meleagris gallopavo* | Wild turkey | 0 | 0 |
|  |  | *Pavo cristatus* | Common peafowl | 2 | 5 |
|  |  | *Phasianus colchicus* | Common pheasant | 0 | 1 |
| *Passeriformes* | *Alaudae* | *Alauda arvensis* | Skylark | 0 | 0 |
|  | *Estrildidae* | *Lonchura punctulata* | Scaly-breasted munia | 3 | 0 |
|  |  | *Padda oryzivora* | Java sparrow | 0 | 0 |
|  | *Fringillidae* | *Carduelis carduelis* | Goldfinch | 2 | 0 |
|  |  | *Carduelis chloris* | Greenfinch | 0 | 0 |
|  |  | *Carduelis flammea* | Common redpoll | 2 | 4 |
|  |  | *Fringilla coelebs* | Chaffinch | 0 | 0 |
|  | *Passeridae* | *Passer domesticus* | House sparrow | 4 | 13 |
|  |  | *Passer montanus* | Tree sparrow | 3 | 11 |
|  | *Pycnonotidae* | *Pycnonotus jocosus* | Red-whiskered bulbul | 4 | 3 |
|  | *Sturnidae* | *Acridotheres tristis* | Common myna | 11 | 16 |
|  |  | *Sturnus vulgaris* | European starling | 5 | 18 |
|  | *Turdidae* | *Turdus merula* | Common blackbird | 12 | 4 |
|  |  | *Turdus philomelos* | Song thrush | 0 | 0 |
| *Struthioniformes* | *Struthionidae* | *Struthio camelus* | Ostrich | 0 | 0 |

**Supplementary Data S2:** Written descriptions of impact levels for each environmental and economic sub-category, as published by Kumschick & Nentwig (2010).

**1 Environmental impact**

**1.1 Herbivory**

0 No impact known or detectable.

1 Similar impact as native species, no major damage to plants reported.

2 Similar impact as native species, recorded negative impact on flora, impact only on abundant species.

3 Generalist herbivore, impact through unselective grazing on plants adapted to grazing, limited damages to trees, minor changes in plant communities with impact on endemic species, negative impact on seed dispersal.

4 Grazing and damage to trees by bark stripping and/or antler rubbing, damage to endemic species, recorded vegetation change reversible.

5 Grazing in areas not adapted to large herbivores, e.g. island ecosystems, high damage through bark stripping and/or antler rubbing, threat to endemic and plant species listed as vulnerable, endangered or critically endangered by IUCN, local extinctions or permanent community changes.

**1.2 Competition**

0 No impact known or detectable.

1 Very low level of competition with at least one native species, exploitation competition.

2 Competition with several native species by exploitation competition, without large impact on affected species or decline of their populations.

3 Competition with several species for food and/or space, interference competition, at least one native species declining.

4 Competition with many native species, several declining in population size, competition for food and/or space, behavioural changes in out-competed species.

5 Competes with species listed as vulnerable, endangered or critically endangered by IUCN, decline of these species, replacement or even extinction of species.

**1.3 Predation**

0 No impact known or detectable.

1 Predation known but negligible, no decline of native species.

2 Predation on several abundant species, without large impact on affected species or decline of their populations.

3 Decline of one to several native species recognized, no changes in food web structure reported.

4 Decline of many species, indirect impact by mesopredator release, changes in the food web.

5 Preys also on endemic or species listed as vulnerable, endangered or critically endangered by IUCN, local extinction.

**1.4 Transmission of diseases to wildlife**

0 No impact known or detectable.

1 Host for non-specific parasites, occasional transmission of more or less harmless diseases to one native species. No population decline in native species.

2 Occasional transmission of more or less harmless diseases, several native species affected. No or only minor population decline in native species.

3 Many native species affected, frequent transmission of more or less harmless diseases or harmful diseases transmitted to one native species. Minor population decline in native species.

4 Transmits harmful diseases to several native species or more or less harmless diseases to endemic or species listed as vulnerable, endangered or critically endangered by IUCN. Moderate population decline in native species.

5 Transmits harmful diseases to many species and/or species listed as vulnerable, endangered or critically endangered by IUCN by direct transmission, decline of these species or extinction.

**1.5 Hybridisation**

0 No impact known or detectable.

1 Hybridisation possible in captivity, but only rarely in the wild.

2 Hybridisation is more common in the wild, no offspring, but constraints to normal mating.

3 Hybridisation is more common, with offspring, but not fertile.

4 Hybridisation common with fertile offspring.

5 Risk of extinction of endangered species.

**1.6 Ecosystem**

0 No impact known or detectable.

1 Slight pollution of water bodies with possible eutrophication. Slight indication of impact on successional processes.

2 Damage of vegetation resulting in bank erosion, eutrophication of water bodies infrequent.

3 Eutrophication of water bodies more frequent, change in aquatic fauna and flora, soil compaction through trampling. Impact on successional processes, infrequently, medium intensity.

4 Severe erosion processes. Eutrophication of water bodies leading to decline of species and/or change in aquatic species composition. Strong impact on successional processes.

5 Eutrophication of water bodies leading to drastic decline of species and/or change in aquatic species composition, endemic species and/or species listed as vulnerable, endangered or critically endangered by IUCN affected. Strong impact on successional processes leads to loss of habitat characteristics, damage of sites of conservation importance.

**2 Economic impact**

**2.1 Agriculture**

0 No impact known or detectable.

1 Only occasional damage to crops or plantations, damage similar to native species.

2 Damage to crops more common.

3 Damage through feeding on crops, occasional threat to stored food, damage exceeds impact of the native fauna.

4 High damage in fields or to stored food, fruit consumption.

5 Complete destruction of fields or plantations, or of stored food by consumption and contamination.

**2.2 Animal production**

0 No impact known or detectable.

1 Occasional competition with livestock.

2 Competition with livestock, transmission of diseases to livestock in the native area, but not yet reported from the area of introduction.

3 Competition more frequent with several livestock species, transmission of diseases reported, but infection rates low. Pollution by droppings on farmland which domestic stock are then reluctant to graze.

4 Transmission of economically important diseases or hybridisation with economically important game animals.

5 Transmission of harmful diseases to or hybridisation with livestock.

**2.3 Forestry**

0 No impact known or detectable.

1 Minor impact through herbivory.

2 Impact through herbivory, minor effect on forest growth, impact on seed dispersal.

3 Constrains forest regeneration through browsing on young trees, damage to plantations, gnawing of bark, damage by causing floods.

4 Moderate to strong damage to mature forest through seed consumption, bark stripping or antler rubbing, death of trees by felling or flooding. Killing trees by defoliating them for nesting material.

5 Very strong damage to mature forest through seed consumption, bark stripping or antler rubbing, death of trees by felling or flooding.

**2.4 Infrastructure**

0 No impact known or detectable.

1 Biological traits and life-style suggest potential damage to infrastructure, but not yet reported.

2 Occasional damage with minor economic losses, e.g. damage to fences.

3 Damage to fences and/or plantations, gnawing electricity cables etc., causing road accidents, nesting on current conductions.

4 High damage with considerable economic costs, damage through burrowing or nesting in buildings, impact through pollution, accumulations of droppings.

5 Considerable damage to flood defence systems, danger to human safety, threat to air safety.

**2.5 Human health**

0 No impact known or detectable.

1 Host of one or more harmless diseases with the possibility of infecting humans, not yet reported.

2 Host of several harmless diseases, indirect transmission or possibility of direct transmission, but only a small percentage of the human population at risk, health hazard from soil and water contamination caused by droppings.

3 Direct infection with one or more harmless diseases, occasionally health thread through bites or other attacks.

4 Direct transmission of several diseases, infection by contaminated food common, host of harmful diseases in the native range, but not yet known from the invaded range. Health thread through bites or other injuries happen more often, rarely fatal.

5 Vector of harmful diseases to humans and/or many diseases frequently transmitted. Health thread through bites or other injuries happen frequently, more often fatal.

**2.6 Human social life**

0 No impact known or detectable.

1 Noise disturbance, only small percentage of human population affected.

2 Noise disturbance more frequent and more intense, slight pollution of recreational areas.

3 Pollution of recreational areas more frequent.

4 Fouling and damage by trampling and overgrazing to rural parks, golf courses and city parks. Recreational value of a habitat or a landscape strongly affected.

5 Pollution of water bodies used for public recreation, which leads to eutrophication and algal blooms, complete loss of recreational value of a habitat or a landscape.

**References**

Kumschick, S. & Nentwig, W. (2010) Some alien birds have as severe an impact as the most effectual alien mammals in Europe. *Biological Conservation*, **143**, 2757-2762.
